# Hydrological dynamics in the China-Mongolia arid region: An integrated analysis of precipitation recycling and water vapor conversion

**DOI:** 10.1016/j.heliyon.2024.e32839

**Published:** 2024-06-13

**Authors:** Ruolin Li, Qi Feng, Yang Cui

**Affiliations:** a Key Laboratory of Ecological Safety and Sustainable Development in Arid Lands, Lanzhou, 730000, China; b Qilian Mountains Eco-Environment Research Center in Gansu Province, Lanzhou, 730000, China; c Key Laboratory of Ecohydrology of Inland River Basin, Northwest Institute of Eco- Environment and Re-sources, Chinese Academy of Sciences, Lanzhou, 730000, China; d Ningxia Institute of Meteorological Sciences, Yinchuan, 75002, China

**Keywords:** Precipitation recycling, Water vapor conversion, Empirical orthogonal functions, China-Mongolia arid region (CMAR), Hydro-meteorological variability, Dynamic recycling model (DRM)

## Abstract

This study examines the atmospheric water cycle dynamics in the China-Mongolia Arid Region (CMAR), a region significantly affected by aridity. By employing a combination of Empirical Orthogonal Function (EOF) analysis, ERA5 reanalysis data, and the Dynamic Recycling Model (DRM), we investigate the spatial and temporal variations in the Precipitation Recycling Ratio (*PRR*) and Precipitable Water Conversion Rate (*PWCR*) over a forty-year period (1979–2021). Our findings reveal that both *PRR* and *PWCR* are generally higher but decreasing in most subregions of CMAR, suggesting a notable contribution of local moisture to precipitation. We also identify an increasing trend in *PRR* across the northwestern subregions and a decreasing trend in other areas. Similarly, *PWCR* exhibits an increasing trend in the northwestern and southern subregions, while decreasing elsewhere, implying a decline in water vapor conversion and recycling efficiency. Furthermore, our EOF analysis uncovers distinct spatial patterns, with dominant modes accounting for significant variances in *PRR* and *PWCR*, correlating with local variations in atmospheric moisture and advective changes. These results underscore the complex interplay between regional topography, atmospheric dynamics, and the hydrological cycle in CMAR. The insights from this study are vital for formulating effective water management strategies and adapting to climate change impacts in arid regions, holding broad implications for environmental science, climate studies, and sustainable resource management. Our findings reveal distinct spatial patterns and contrasting trends in precipitation recycling and water vapor conversion across the subregions of CMAR. This heterogeneity underscores the importance of conducting analyses at finer spatial scales to avoid contradictory conclusions that can arise from topographic influences when treating CMAR as a single unit. Future studies should focus on smaller subregions to accurately capture the intricacies of the water cycle in this topographically complex arid region.

## 1 Introduction

This study investigates the atmospheric water cycle in the China-Mongolia arid region (CMAR), focusing on precipitation recycling, water vapor conversion, and their interactions with atmospheric water vapor dynamics. The CMAR, covering parts of northern China and southern Mongolia, experiences low annual rainfall and high evaporation rates [1,2]. The region's climate is significantly influenced by these factors, with most precipitation occurring as convective rainfall during summer [3,4].

Precipitation recycling in CMAR plays a key role in the region's annual rainfall [5,6]. This process involves evaporated water traveling through the atmosphere before returning as precipitation, either locally or in adjacent areas [7,8]. Understanding the dynamics of this process is crucial due to its substantial impact on regional climate variability. Numerous studies highlight the importance of precipitation recycling (PR) in maintaining the water balance in areas like the CMAR. For example, research by Li and Wang [5] quantified PR in the CMAR and found that local and regional recycling processes contribute to about 10 % of the CMAR's annual rainfall, emphasizing the importance of understanding the water cycle dynamics in such dry areas. Similarly, Li et al. [9] shows the precipitation recycling ratio in North Africa and China-Mongolia is stronger in summer but weaker in winter. Nevertheless, research by Wang et al. [10] presents large-scale ecological restoration programs have a positive effect on local moisture cycle and precipitation.

Another vital aspect of the water cycle in arid regions like CMAR is the conversion of atmospheric water vapor into precipitation, known as precipitable water conversion [11]. This process is essential for rainfall formation in areas where the moisture source for rainfall is limited [12]. Wei et al. [13] explored factors that influence the efficiency of rainfall formation in semi-arid region, suggesting that a better understanding of processes that lead to rain can improve water resource management strategies.

Previous studies have primarily focused on the quantitative analysis of precipitation recycling and precipitable water conversion, highlighting their crucial roles in the overall water cycle [[14], [15], [16], [17]]. Dominguez et al. [14] developed a dynamic precipitation recycling model that incorporates changes in moisture storage, allowing for analysis at various temporal scales. Zhang et al. [15] used a precipitation recycling process model to analyze the contribution of local moisture to total precipitation in the middle and lower reaches of the Yangtze River. Rozos and Makropouos [16] investigated the combined benefits of water recycling technologies in the urban water cycle, emphasizing the potential of sustainable, water-aware technologies to reduce environmental pressures. However, Goessling and Reick [17] cautioned that moisture recycling estimates may not fully capture the consequences of land-use change for precipitation, as other mechanisms such as local coupling and atmospheric circulation can also play significant roles.

Further, a range of studies have explored the complex relationship between hydrological-meteorological variables like PR and PWC. Hua et al. [18] found that the precipitation recycling ratio increased in arid and semi-arid regions of China, particularly before the 1990s, and identified a positive correlation between precipitation efficiency and recycling ratio. Results from Zhang et al. [15] showed the precipitation recycling rate is important to evaluate the contribution of different water vapor sources. Keys et al. [19] quantified the variability of the precipitationshed boundary, which determines the sources of evaporation for a region's precipitation and found that a core precipitationshed exists with a pulsing of evaporation. Zang et al. [20] analyzed the spatiotemporal patterns of key hydrological variables in China, showing that runoff and infiltration were mainly influenced by precipitation, while actual evapotranspiration was constrained by a combination of precipitation and energy. These studies collectively highlight the importance of considering the dynamic and spatially variable nature of these processes in understanding the water cycle.

Building on the existing research and analytical findings, our study utilizes the high temporal-spatial resolution dataset and dynamic statistical methods (the DRM model and empirical orthogonal functions, EOFs) to analyze the atmospheric hydrological characteristics of CMAR over the past four decades. The aim is to investigate the spatiotemporal variability of the water cycle in different regions of CMAR and to explore the dynamic relationships between them. The paper is structured as follows: Section 2 provides an overview of the data sources and methodologies used in our study. Sections 3 presents detailed findings on precipitation and evaporation, supplemented by comprehensive analyses of water vapor transport and transformation. Section 4 focuses on exploring the relationship between the precipitation recycling ratio (*PRR*), precipitable water conversion rate (*PWCR*) and atmospheric water vapor dynamics. Employing Empirical Orthogonal Function (EOF) techniques, we examine the spatial patterns of *PRR* and *PWCR* and discuss the potential impacts of *PRR* and *PWCR* on precipitation. Section 5 summarizes the key conclusions from our research. Finally, Section 6 discusses the main outcomes and outlines directions for future investigations in this field.

## 2 Material and methods

### 2.1 Hydro-meteorological data

#### 2.1.1 Meteorologic data

In this study, we harnessed the ERA5 reanalysis dataset from the European Centre for Medium-Range Weather Forecasts (ECMWF), providing a comprehensive suite of climatic variables from 1979 to 2021. This dataset, with its high spatial resolution of 31 km and hourly temporal granularity, incorporates observational data from a myriad of sources—satellites, weather balloons, aircraft, maritime vessels, and terrestrial weather stations—via an advanced hybrid 4D-Var assimilation process [21]. Precipitation (*P*) and evaporation (*E*) rates, expressed in mm/day, are derived from the ERA5-Land reanalysis, interpolated to a 0.25° grid. Precipitable water (*PW*) values are obtained from the ERA5 single-level reanalysis, with the same spatial resolution and in units of kg/m2. Zonal (*u*) and meridional (*v*) wind components, vital for understanding atmospheric circulation, are also sourced from ERA5 reanalysis, covering the same period and spatial resolution in m/s at multiple atmospheric pressure levels.

The ERA5 dataset's increased spatial and vertical resolution, the broader range of observational data assimilation, and the enhanced modeling techniques mark a significant improvement over previous iterations, offering more precise measurements and uncertainty estimates for each variable [[21], [22], [23]]. The use of this dataset is crucial for examining extended climate trends and variations across Earth's subsystems.

While ERA5 represents a significant advancement in reanalysis datasets, it is important to acknowledge its limitations, especially when considering precipitation in arid regions like CMAR. Precipitation estimates in re-analyses are derived from the model's physical parameterizations and are not directly constrained by observations, which can lead to biases and uncertainties [21]. In arid regions, the scarcity of in-situ observations and the challenges in accurately representing convective processes and land-atmosphere interactions can further contribute to these uncertainties [24]. Despite these limitations, ERA5 remains one of the most reliable and widely used reanalysis datasets for studying hydro-meteorological processes. However, the interpretation of our results should be made with these caveats in mind, and future studies could benefit from the integration of additional observational data sources to validate and complement the ERA5-based findings.

#### 2.1.2 Integer vapor transport (*IVT*)

*IVT*, a crucial measure in this study, is computed using specific humidity and wind components at different atmospheric pressure levels [25,26]. The formula employed is

(1) $$IVT=\sum_{p=P_{s}}^{P_{t}}\left[\left(\vec{u_{p}}\cdot q_{p}\right)+\left(\vec{v_{p}}\cdot q_{p}\right)\right]$$

where *Ps* and *Pt* represent surface and top pressures, respectively, ${\vec{u}}_{p}$ and ${\vec{v}}_{p}$ are the zonal and meridional wind components at pressure *p*. $q_{p}$ is the specific humidity at pressure p. The unit is kg/(m·s). The data of $P_{s}$*,* $\vec{u_{p}}$, $\vec{v_{p}}$ and $SH_{p}$ are from ERA5 reanalysis data. The data is from 1979 to 2021. The data is interpolated to 0.25°. In this research, the top pressure level (*Pt*) is selected as 300 hPa, $P_{s}$ is 1000 hPa and the pressure interval as 1000, 975, 950, 925, 900, 875, 850, 825, 800, 775, 750, 700, 650, 600, 550, 500, 450, 400, 350, 300 hPa.

While ERA5 provides direct estimates of *IVT*, we chose to calculate it using Equation (1) to maintain consistency with previous studies [27,28] and to have flexibility in selecting the vertical levels for integration based on our research objectives. Further, A comparison between the *IVT* calculated using Equation (1) and the direct ERA5 estimates is provided in Supplementary Fig. S1. The close agreement demonstrates that our *IVT* calculation method is robust and captures the key influences of CMAR's complex terrain on water vapor transport, making it well-suited for this study.

#### 2.1.3 Precipitable water conversion rate

The precipitable water conversion rate (*PWCR*) is the ratio of the total atmospheric water vapor that is converted into precipitation, measuring how efficiently the water vapor in a column of air is turned into rain or snow [29,30].

*PWCR* can be written as:

(2) $$PWCR=\frac{P}{PW}\cdot 100\%$$

where *P* is the precipitation and *PW* is the precipitable water.

The precipitable water is defined as

(3) $$PW=\int_{P_{s}}^{P_{t}}\bar{q}\frac{dp}{g}$$

where $P_{s}$ and $P_{t}$ are the surface and top pressure, respectively, and *q* is the specific humidity. It is the precipitable water in the total column from the surface to the top of the atmosphere.

#### 2.1.4 The Atmospheric Water Vapor Continuity Equation

The Atmospheric Water Vapor Continuity Equation, which states that the time rate of change of specific humidity due to local variations plus the rate of change due to the transport of water vapor by atmospheric motions equals the precipitation rate plus the evaporation rate plus other processes that affect the water vapor content [31,32].

(4) $$\frac{\partial q}{\partial t}+\left(\partial qu/\partial x+\partial qv/\partial y\right)=E-P-C$$

The change of specific humidity with time $\frac{\partial q}{\partial t}$ plus the change due to atmospheric motion (advection, *∂qu/∂x* + *∂qv/∂y*) equals the precipitation rate *P* plus the evaporation rate *E* plus other processes *C* that affect the water vapor amount and can be neglected.

The application of the Atmospheric Water Vapor Continuity Equation in this study is based on several assumptions and has certain limitations. Firstly, the equation assumes that the atmosphere is well-mixed, implying that the ratio of advected to evaporated water vapor is equivalent to the ratio of advected to recycled precipitation. While this assumption simplifies the analysis, it may not always hold true in real-world conditions, particularly in regions with complex topography or strong vertical mixing [14].

Secondly, the equation assumes that changes in atmospheric moisture storage are negligible at monthly or longer time scales. While this assumption is generally valid for long-term analyses, it may introduce uncertainties when considering shorter time periods or regions with significant seasonal variations in moisture storage.

Furthermore, the accuracy of the results obtained from this equation depends on the quality and resolution of the input data, such as specific humidity, wind components, and precipitation and evaporation rates. Any uncertainties or biases in these input variables can propagate into the calculated water vapor changes and affect the overall conclusions.

Despite these limitations, the Atmospheric Water Vapor Continuity Equation remains a valuable tool for understanding the dynamics of water vapor in the atmosphere and its relationship to precipitation and evaporation processes. The results obtained from this approach should be interpreted in light of these assumptions and limitations, and future studies could explore ways to refine the methodology and address these challenges.

#### 2.1.5 Precipitation recycling

The delineation of CMAR and its subregions was based on a modeling approach using the Dynamic Recycling Model (DRM) developed by Dominguez et al. [14], which is a well-established method for quantifying precipitation recycling processes. *PRR* in this study is calculated using DRM, which delineates advected and recycled water vapor in the atmosphere [33]. *PRR* is defined as the ratio of locally evaporated water (*Pr*) that contributes to *P*, expressed as:

(5) $$\rho =\frac{P_{r}}{P}$$

The DRM, based on the local water balance, computes this ratio by considering the total *PW* and its changes due to *E* and *P*, with the relationship:

(6) $$\frac{\partial PW}{\partial t}+\frac{\partial \left(PWu\right)}{\partial x}+\frac{\partial \left(PWv\right)}{\partial y}=E-P$$

where *u* and *v* represent the horizontal wind components.

Under the assumption that the atmospheric column is well mixed, the DRM posits that the ratio of advected to evaporated water vapor is equivalent to the ratio of advected to recycled precipitation:

(7) $$\frac{PW_{a}}{PW_{r}}=\frac{P_{a}}{P_{r}}$$

Moreover, at monthly or longer time scales, changes in atmospheric moisture storage are deemed negligible.

The DRM separates Eq. (6) into advected and recycled components, represented by Eq. (7), while recycling mass conservation is given by:

(8) $$PW\frac{\partial \rho }{\partial t}+PWu\frac{\partial \rho }{\partial x}+PWv\frac{\partial \rho }{\partial y}=E\left(1-\rho \right)$$

The solution to these equations is found in a transformed coordinate system:

(9) χ=x−utξ=y‐vtτ=t

where the recycling ratio R in this new system is:

(10) $$R=1-\exp\left(-\int_{0}^{\tau }\frac{\epsilon }{\omega }\partial \tau^{\prime }\right)$$

with *ε* and *ω* being the evaporation and water vapor content, respectively. The full derivation is detailed in Refs. [14,34].

This methodological approach enables a nuanced analysis of CMAR's hydrological processes, considering both the local contributions to precipitation and the broader atmospheric water vapor dynamics.

### 2.2 Anomaly calculations

In the realm of climatology and hydrology, anomaly calculations are pivotal for identifying deviations from established averages, offering insights into climatic trends and variability [35,36]. This methodology consists of three primary steps:

1. Baseline Determination: A typical reference period spans 30 years to define a ‘normal’ state.
2. Baseline Average Calculation: Averages for specific intervals (e.g., monthly, seasonally) within this period are computed to serve as a benchmark.
3. Anomaly Computation: Anomalies for each interval are calculated by the formula:

(11) A=O−B

where *A* represents the anomaly, *O* is the observed value, and *B* is the baseline average.

4. Anomaly percentage: A measure of the relative deviation of a variable from its baseline average, expressed as a percentage. It is calculated as:

(12) $$AP=\frac{\left(O-B\right)}{B}$$

where *AP* is anomaly percentage.

These calculations enable the standardization of comparisons across various temporal and spatial scales. Positive anomalies indicate values above the average, while negative anomalies reflect values below it, essential for interpreting data within the broader context of climate studies.

### 2.3 Empirical orthogonal functions (EOFs)

This study employs Empirical Orthogonal Functions (EOFs) to investigate variability within CMAR's hydro-meteorological datasets. EOF analysis reduces complex datasets to principal modes, clarifying dominant variability patterns [37]. This statistical approach isolates significant spatial and temporal trends, contributing to our understanding of CMAR's water cycle dynamics [38].

Applying EOFs to ERA5-derived variables like precipitation, evaporation, and vapor transport helps unravel the main factors influencing CMAR's hydrology. The EOFs decompose a data matrix *X* into spatial patterns (*U*) and temporal coefficients (*V*), expressed as *X* = *U⋅VT*, where *VT* is the transpose of *V*. The leading EOFs, which are orthogonal and represent the largest variance, offer insights into the most influential hydro-meteorological patterns [39].

The significance of EOFs lies in their interpretative power, correlating with climatic factors to elucidate water cycle dynamics. By focusing on the highest-variance EOFs, we identify and interpret the primary modes impacting CMAR's hydrology, bridging data analysis with climatological understanding.

### 2.4 Study area

**Fig. 1:**
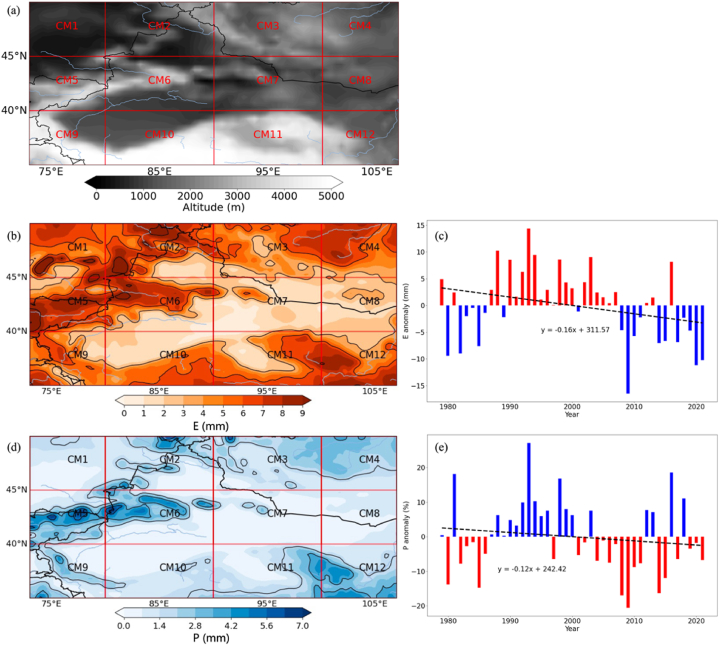
Topographical Map of the China-Mongolia Arid Region (CMAR) with the delineated sub-regions (CM1 to CM12) (a), and Hydrological Patterns and Anomalies During Summer in CMAR (1979–2021) (b) Displays the distribution of evaporation during the summers of 1979–2021. (c) Shows changes in evaporation anomalies for CMAR from 1979 to 2021. (d) Maps the distribution of precipitation over the summers of 1979–2021. (e) Illustrates the percent change in precipitation anomalies for the June–August period from 1979 to 2021.

CMAR encompasses a large area in Northern China and Southern Mongolia, distinguished by its varied topography and climate. Fig. 1 shows a topographical map of CMAR, which is divided into twelve sub-regions (CM1 to CM12) for an in-depth analysis of the atmospheric water cycle. CMAR's geographical boundaries extend from 35°N to 50°N latitude and from 73°E to 107°E longitude, with each sub-region's specific coordinates detailed in Table 1.

**Table 1:** Sub-region latitude-longitude range.

Sub-region	Latitude	Longitude	Major Geographic Features	Altitude
CM 1	45∼50°N	73∼80°E	Zaysan Lake	High
CM 2	45∼50°N	80∼90°E	Altai Mountains	High
CM 3	45∼50°N	90∼100°E	Mongolian Plateau	Low
CM 4	45∼50°N	100∼107°E	Gobi Desert	Low
CM 5	40∼45°N	73∼80°E	Tian Shan Mountains	High
CM 6	40∼45°N	80∼90°E	Tarim Basin	Low
CM 7	40∼45°N	90∼100°E	Taklamakan Desert	Low
CM 8	40∼45°N	100∼107°E	Gobi Desert	Low
CM 9	35∼40°N	73∼80°E	Kunlun Mountain	High
CM 10	35∼40°N	80∼90°E	Altyn-Tagh	High
CM 11	35∼40°N	90∼100°E	Qilian Mountains	High
CM 12	35∼40°N	100∼107°E	Loess Plateau	High

CMAR was divided into twelve subregions (CM1 to CM12) based on a combination of latitudinal and longitudinal boundaries. The latitudinal divisions were set at 5° intervals, creating three main latitudinal bands: 45–50°N, 40–45°N, and 35–40°N. The longitudinal divisions were then applied within each latitudinal band, with the aim of creating subregions that capture the spatial variability of hydrological processes while maintaining a manageable number of units for analysis.

The longitudinal boundaries were set at 73°E, 80°E, 90°E, 100°E, and 107°E, resulting in four subregions within each latitudinal band. These longitudinal divisions were chosen to align with major geographical features and climatic gradients within CMAR, such as mountain ranges, basins, and transitions between different precipitation regimes [2,3]. By combining the latitudinal and longitudinal boundaries, we obtained the twelve subregions (CM1 to CM12) used in this study, each representing a unique combination of geographical and climatic characteristics.

The northernmost subregions (CM1-CM4) are characterized by the Altai Mountains in the west and the Mongolian Plateau in the east. The Irtysh River flows through CM1, while the Selenga River and its tributaries drain CM2 and CM3. The Gobi Desert dominates the landscape in CM4.

The central subregions (CM5-CM8) feature the Tian Shan Mountains in the west (CM5) and the Gobi Desert in the east (CM8). The Tarim Basin and the Taklamakan Desert are the main geographical features in CM6 and CM7, with the Tarim River being the primary water source.

The southernmost subregions (CM9-CM12) include the Qilian Mountains and the Hexi Corridor in the west (CM9), the Loess Plateau in the center (CM10 and CM11), and the North China Plain in the east (CM12). The Yellow River and its tributaries are the main water sources in these subregions, with the Qinghai Lake being a prominent water body in CM9.

Altitude variation across CMAR, as indicated by the shading on the map (Fig. 1a), significantly influences the regional climate and water cycle dynamics. Regions at higher altitudes generally experience lower temperatures and distinct precipitation patterns compared to lower areas. This subdivision into sub-regions enables a comprehensive assessment of spatial variations in factors such as precipitation recycling and water vapor conversion.

The map's blue lines mark major rivers and lakes within CMAR (Fig. 1a), integral components of the regional water cycle. These water bodies, together with the altitude variation, impact local water vapor content and precipitation patterns, influencing key water cycle metrics like *PRR* and *PWCR*. The inclusion of major rivers and lakes in the map is essential from a meteorological perspective. A high number of water bodies in a region increases the potential evaporative surface, leading to an increase in local evaporation [40,41]. This locally evaporated moisture can then contribute to the formation of precipitation in the same area, a process known as local moisture recycling [10,42]. In arid regions like CMAR, where moisture sources are limited, these water bodies can play a crucial role in the regional water cycle by providing a source of local moisture [43]. The evaporation from these surfaces influences the spatial distribution of atmospheric water vapor, which in turn affects the patterns of precipitation recycling and water vapor conversion [9]. Therefore, considering the location and extent of these water bodies is important for understanding the hydrological dynamics within CMAR.

This study's segmentation of CMAR into sub-regions facilitates an analysis of spatial and temporal variations in the water cycle. By focusing on these divisions, the research aims to uncover localized patterns in atmospheric moisture movement and recycling. Such insights are crucial for effective water resources management and formulating strategies for climate change adaptation within the region.

## 3 Characteristics of the water cycle within CMAR

In this section, we comprehensively analyze the characteristics of the water cycle within CMAR, focusing on the intricacies of precipitation and evaporation patterns, as well as the dynamics of water vapor transport, recycling, and transformation. The analysis encompasses both temporal and spatial dimensions, delineating significant trends and spatial disparities in precipitation, evaporation, and water vapor characteristics.

### 3.1 Analysis of precipitation and evaporation patterns

Empirical observations and prior research indicate that significant evaporation [44], precipitation [45,46], as well as agricultural activities and plant growth [47,48] in the region, predominantly occur during the summer months (June, July, August - JJA). Consequently, this study focuses primarily on this period.

The decision to focus on the summer months (June, July, August - JJA) in this study is justified by the significant portion of annual precipitation occurring during this season in CMAR, driven by the East Asian Summer Monsoon (EASM) and westerly circulation [49,50]. These atmospheric circulation patterns bring moisture to the region, leading to increased convective activity and precipitation.

Moreover, high solar radiation and temperatures during summer enhance evaporation rates, contributing to local moisture recycling and influencing convective precipitation formation. This makes summer a critical period for understanding hydrometeorological processes in arid regions like CMAR.

While acknowledging the importance of considering the full annual cycle, focusing on the summer months allows us to capture the most important period for water cycle dynamics in the region. Future studies could explore the hydrometeorological processes throughout the year for a more comprehensive understanding of CMAR's water cycle.

Fig. 1 (b to e) displays the average spatial distribution of evaporation and precipitation during the summer months in CMAR from 1979 to 2021. The spatial analysis of evaporation, as shown in Fig. 1b, indicates a gradient in evaporation rates, more pronounced in regions depicted in darker shades of orange to red. A comparative analysis of evaporation and precipitation distributions, in conjunction with topographical data from Fig. 1, indicates a clear relation. Sub-regions such as CM5, CM6, and CM9, characterized by their specific topographical features, exhibit higher evaporation rates, ranging from 2.5 to 3.2 mm day^−1^. These regions are predominantly at lower altitudes, suggesting a relationship with temperature and solar radiation. Sub-regions such as CM5, CM6, and CM9, characterized by their specific topographical features, exhibit higher evaporation rates.

In contrast, the distribution of precipitation in CMAR, depicted in Fig. 1d–is more uniform. However, areas represented in darker blue denote higher precipitation levels. These are typically higher altitude areas where orographic effects influence rainfall patterns. Sub-regions like CM1, CM4, and CM12, positioned at higher elevations, tend to receive more precipitation due to interception of moisture-rich air.

This spatial analysis highlights the significant influence of topography on the distribution patterns of evaporation and precipitation within CMAR, underscoring altitude as a key factor in these observed variations.

Fig. 1c and (e) presents an analysis of evaporation and precipitation anomalies in the CMAR covering the period from 1979 to 2021. These anomalies, representing deviations from the long-term average, provide insights into interannual variability and general trends.

In Fig. 1c, the annual deviations from the mean evaporation rate are displayed. Years with evaporation rates above the average are marked in red, signifying positive anomalies, while years with rates below the average are in blue, indicating negative anomalies. A dashed line in this figure illustrates a negative linear trend, indicative of a consistent decrease in evaporation within CMAR over time. This trend may be attributable to regional climatic changes, potentially related to broader global climate patterns or changes in local land use [51].

Regarding precipitation anomalies percentage, as shown in Fig. 1e, the representation is similar. Positive deviations from the average precipitation are indicated in blue, while negative deviations are represented in red. Here too, the trend line shows a negative direction, a dashed line in this figure illustrates a negative linear trend of −0.02 mm day^−1^ per decade (p < 0.05), indicative of a consistent decrease in evaporation within CMAR over time. suggesting a gradual decrease in precipitation over the analyzed period. This reduction in precipitation could be due to alterations in atmospheric circulation or impacts of human activities on the regional climate.

The linear trend analysis of both evaporation and precipitation in CMAR points towards increasing water scarcity and ecological pressures. The simultaneous reduction in these two key variables implies an intensification of arid conditions, potentially affecting the long-term sustainability of water resources in the region.

**Fig. 2:**
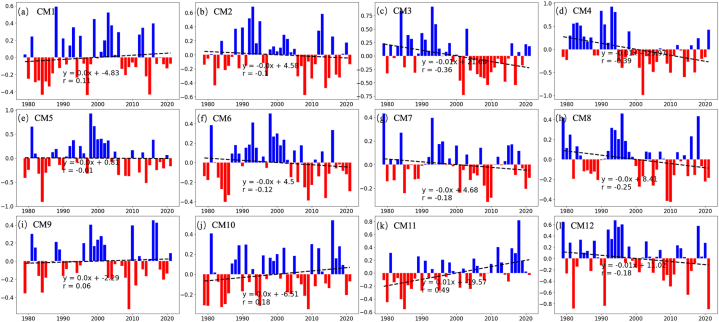
Variations in June–August Precipitation Anomalies Percentage Across CMAR Subregions (1979–2021) Panels (a–l) present the annual precipitation anomalies percentage for twelve subregions (CM1-CM12) in CMAR, with blue indicating positive anomalies, red for negative anomalies, and dashed lines showing the linear trend within each subregion, depicting the fluctuation in precipitation across four decades. (For interpretation of the references to color in this figure legend, the reader is referred to the Web version of this article.)

The spatial distribution and temporal evolution analysis in CMAR reveals uneven patterns of precipitation and evaporation, highlighting the need to analyze variation trends across different subregions. Fig. 2 illustrates the annual precipitation anomalies for twelve subregions (CM1-CM12) in CMAR, spanning from 1979 to 2021. These anomalies, calculated as deviations from the long-term mean, are depicted with positive anomalies in blue bars and negative anomalies in red bars. Subregions CM3 (Fig. 2c), CM4 (Fig. 2d) and CM12 (Fig. 2l) exhibit more pronounced negative trends, with precipitation anomalies decreasing by 0.15–0.25 mm day^−1^ per decade (p < 0.01). Linear trends over the four-decade period are represented by dashed lines across the charts of each subregion. While subregions CM5 (Fig. 2e), CM7 (Fig. 2g), CM8 (Fig. 2h), and CM9 (Fig. 2i) did not exhibit significant changing trends.

The data demonstrate varied trends in precipitation anomalies among the subregions, reflecting the diverse climatic and topographical influences within CMAR. Subregions such as CM2 (Fig. 2b) and CM6 (Fig. 2f) show minor negative trends in precipitation. In contrast, subregions CM3 (Fig. 2c), CM4 (Fig. 2d) and CM12 (Fig. 2l) exhibit more pronounced negative trends. On the other hand, CM1 (Fig. 2a), CM10 (Fig. 2j) and CM11 (Fig. 2k) present a positive trend in precipitation anomalies. These trends indicate heterogeneity in precipitation changes across CMAR, with the central and eastern areas experiencing a decrease, and the northwestern and southern regions observing an increase in summer precipitation relative to the long-term average.

**Fig. 3:**
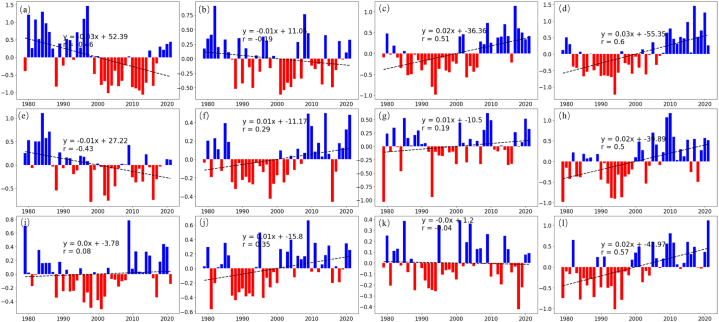
Same with Fig. 4 but Evaporation anomalies.

Fig. 3 presents the variability and long-term trends in evaporation anomalies for the subregions CM1 through CM12 of CMAR from 1979 to 2021. The panels (a-l) correspond to each subregion, with positive evaporation anomalies shown in blue and negative anomalies in red. Linear trend lines are overlaid as dashed lines to illustrate the trend for each subregion over the study period.

The analysis depicted in the figures highlights the variability of evaporation anomalies across the subregions of CMAR, reflecting the influence of interannual climatic changes. This analysis is essential for understanding the dynamics of the water cycle within CMAR.

In subregions CM1 (Fig. 3a), CM2 (Fig. 3b), and CM5 (Fig. 3e), there is a general negative trend in evaporation anomalies, suggesting a long-term reduction in evaporation. This trend could be linked to alterations in land surface characteristics or atmospheric conditions. Conversely, subregions CM9 (Fig. 3i) and CM11 (Fig. 3k) show variable evaporation anomalies, with no consistent trend evident over time. Notably, subregion CM3 (Fig. 3c), CM4 (Fig. 3d), CM6 (Fig. 3f), CM7 (Fig. 3g), CM8 (Fig. 3h), CM10 (Fig. 3j) and CM12 (Fig. 3l) exhibits a positive trend, indicating an increase in evaporation anomalies during the period analyzed.

A comparison between Fig. 2, Fig. 3 reveals that most subregions exhibit opposing trends in evaporation and precipitation. Subregions with reduced precipitation (CM3, 4, 6, 7, 8, 12) show increased evaporation, whereas those with increased precipitation (CM1, 5) display decreased evaporation. Subregions CM2, 9, and 11 do not show significant trends. Overall, the different subregions of CMAR display opposing trends in evaporation and precipitation, typically characterized by decreasing precipitation and increasing evaporation. The observed spatial and temporal variability in precipitation and evaporation anomalies across the figures underscores that, with reduced precipitation, local water vapor plays a more significant role in the regional water cycle of CMAR.

The observed decline in evaporation in certain subregions of CMAR, contrary to the global trend of increasing evaporation due to warming, can be attributed to the interplay of multiple factors. Firstly, changes in land use and vegetation cover can significantly influence evapotranspiration rates [51]. Subregions that have experienced deforestation, overgrazing, or desertification may exhibit reduced evaporation due to the loss of moisture-transpiring vegetation [10,52]. Secondly, alterations in precipitation patterns, as observed in some areas of CMAR, can directly impact soil moisture availability [44,53], thereby limiting evaporative processes. Additionally, regional variations in atmospheric circulation patterns and moisture advection could contribute to the observed spatial heterogeneity in evaporation trends within CMAR [54].

### 3.2 Dynamics of water vapor transport and transformation

This section delves into the dynamics of water vapor transmission and its transformations within CMAR, providing insight into the characteristics of the region's water cycle.

**Fig. 4:**
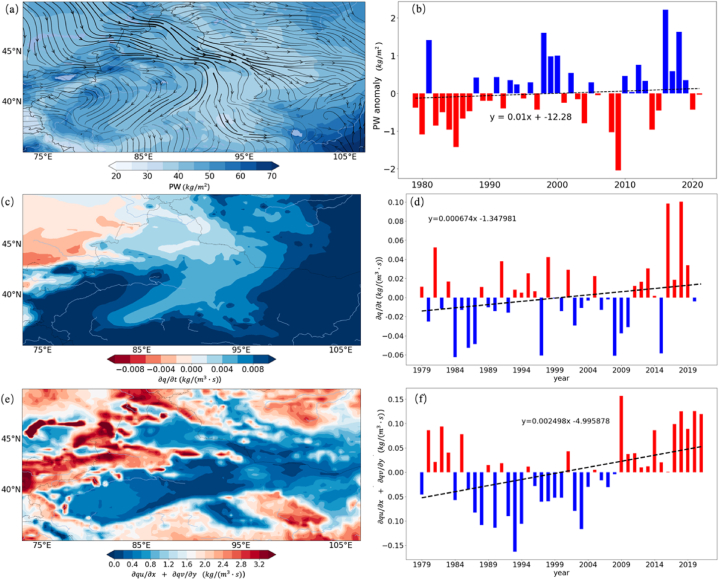
Dynamics of Water Vapor and Anomalies in CMAR (1979–2021) (a) Illustrates precipitable water (*PW*) variability across CMAR using a color gradient for quantity and streamlines for *IVT*, indicating moisture levels and movement. (b) Shows a bar graph of annual *PW* anomalies, with blue for positive and red for negative, highlighting interannual moisture variability. (c) Depicts spatial local water vapor changes (*∂q/∂t*), with intensity reflecting change magnitude. (d) Displays temporal local water vapor changes, with red bars for increases and blue for decreases. (e) Maps the spatial advective water vapor changes (*∂qu/∂x* + *∂qv/∂y*), indicating moisture movement. (f) Details temporal advective water vapor changes, with red signaling increased transport and blue denoting decreased transport, showing the yearly advection trends. (For interpretation of the references to color in this figure legend, the reader is referred to the Web version of this article.)

Fig. 4a displays a visual representation of precipitable water levels and *IVT* in CMAR. The intensity of the fill color in the figure corresponds to the concentration of atmospheric moisture, with darker shades indicating areas of higher precipitable water content. These areas often overlap with regions having significant altitude variations, such as valleys and basins, which were previously identified as zones of higher local evaporation. This overlap suggests a notable influence of evaporation from terrestrial water sources on atmospheric moisture.

The figure also features streamlines representing *IVT* flow, underscoring the role of westerly winds in transporting moisture across CMAR. The interaction between these winds and the regional topography, especially mountain ranges, influences the flow, potentially leading to orographic precipitation or the development of rain shadows.

The concurrence of zones with higher precipitable water and areas of increased evaporation, as identified in earlier evaporation analyses (Fig. 1b and c and Fig. 3), indicates a possible feedback mechanism. In this mechanism, evaporation contributes to local moisture levels, which may subsequently influence precipitation patterns in the region. Meanwhile, Fig. 4 (b) illustrates the specific variability in *PW* over time, showing only a slight increasing trend.

In order to further analyze the dynamics of water vapor in CMAR, this study examines the two primary dynamic factors determining the individual changes in water vapor content (*dq/dt*): local water vapor change (*∂q/∂t*) and advective water vapor change (*∂qu/∂x* + *∂qv/∂y*). Local water vapor change (*∂q/∂t*) refers to changes in water vapor due to local atmospheric variations, while advective water vapor change (*∂qu/∂x* + *∂qv/∂y*) results from the advection of water vapor. Fig. 4c-d displays the spatial and temporal variations of these factors.

In Fig. 4, panel (c) presents the spatial variability of local water vapor changes, likely arising from a combination of evaporation, transpiration, and condensation within CMAR's varied landscapes. Panel (d) illustrates the temporal variation in local water vapor content. Here, red bars indicate years with an increase in local water vapor, possibly due to increased evaporation rates, while blue bars represent years with a decrease, potentially linked to higher precipitation or reduced evaporation rates.

For advective water vapor change (*∂qu/∂x* + *∂qv/∂y*), panel (e) shows the spatial pattern of this change, with color gradients depicting the movement of moist air masses influenced by the region's prevailing wind patterns. Panel (f) indicates the inter-annual variability in advective water vapor change. Positive anomalies suggest periods of increased moisture transport into CMAR, while negative anomalies indicate reduced moisture inflow.

The spatial patterns in panels (c) and (e) indicate a relationship between altitude, local hydrological conditions, and advective moisture processes. Higher altitude areas might experience increased local water vapor changes, possibly due to orographic effects. The interaction between topography and atmospheric circulation, influencing the spatial distribution of water vapor, can result in orographic rainfall in mountainous regions and dry conditions in leeward areas.

The temporal patterns observed in panels (b), (d), and (f) reflect CMAR's response to a combination of internal climatic factors and external atmospheric influences. These patterns display notable periodicity, with the variability cycles of advective water vapor change (*∂qu/∂x* + *∂qv/∂y*) spanning approximately 5–10 years, a duration that exceeds those observed for precipitable water (*PW*) and local water vapor change (*∂q/∂t*). Such observations underscore the significant role of large-scale atmospheric conditions in shaping the region's water vapor dynamics.

Furthermore, it is important to note that long-term trends for *PW*, (*∂q/∂t*), and (*∂qu/∂x* + *∂qv/∂y*) all show an increasing tendency, indicating a regional trend toward greater atmospheric moistening and accelerated moisture transport. This suggests that the atmosphere in the area is exhibiting characteristics of increased humidity and enhanced vapor transport over time.

### 3.3 PRR and PWCR in CMAR

Having examined the spatial and temporal characteristics of evaporation, precipitation, and water vapor dynamics in CMAR, the focus now shifts to quantifying and analyzing *PRR* and *PWCR* within the region. This analysis will build on the understanding of local and advective water vapor changes to elucidate the role of recycled precipitation in the regional water cycle and the efficiency of water vapor conversion into precipitation.

#### 3.3.1 Precipitation recycle ratio (*PRR*)

Having examined the spatial and temporal characteristics of evaporation, precipitation, and water vapor dynamics in CMAR, the focus now shifts to quantifying and analyzing *PRR* and *PWCR* within the region. This analysis will build on the understanding of local and advective water vapor changes to elucidate the role of recycled precipitation in the regional water cycle and the efficiency of water vapor conversion into precipitation.

**Fig. 5:**
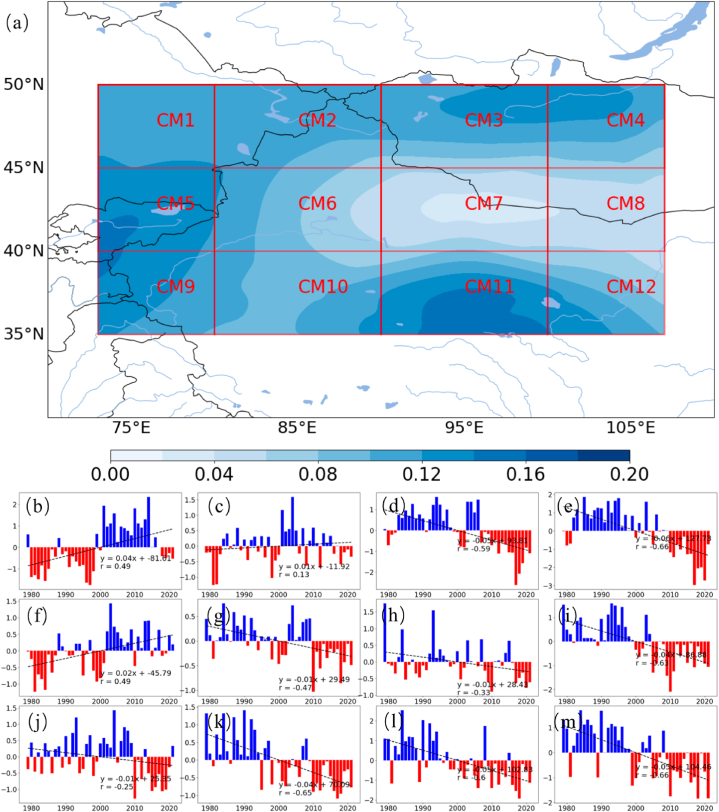
Spatial and Temporal Variability of Precipitation Recycling Ratio (*PRR*) in CMAR (a) shows *PRR* spatial distribution across CMAR subregions. (b–m) *PRR* anomaly changes from 1980 to 2020 in each subregion, with blue bars representing above-average and red bars below-average recycling. (For interpretation of the references to color in this figure legend, the reader is referred to the Web version of this article.)

Fig. 5 offers a comprehensive analysis of *PRR* in CMAR. Fig. 5a displays the spatial distribution of *PRR*, highlighting the contribution of locally recycled moisture to total precipitation in the twelve subregions. The color gradient in the figure corresponds to different levels of *PRR*, with darker shades indicating higher ratios. This spatial distribution is influenced by the region's physical landscape and climatic conditions. Notably, subregions such as CM5 and CM9, characterized by significant water bodies or varied terrain, show elevated *PRR* levels. In the highest altitude subregions, CM10 to CM12, *PRR* is also relatively high, reaching around 0.16, suggesting effective moisture recycling potentially influenced by local environmental conditions.

Fig. 5(b–m) present the annual anomaly changes in *PRR* for each subregion from 1979 to 2021. The bar charts in these panels depict fluctuations in *PRR* over the years. Blue bars signify years with higher-than-average recycling ratios, while red bars indicate lower-than-average years. These variations likely result from changes in land use, vegetation patterns, and climatic factors such as temperature and wind patterns.

The data indicate a general decreasing trend in *PRR* across most subregions, particularly in the eastern and southern subregions (CM4, CM8, CM9-12), where evaporation trends are increasing. This suggests that in these areas, locally evaporated water vapor is not efficiently converted into precipitation but is instead transported to other regions. This phenomenon may be related to the region's terrain and climate, as well as human activities.

The analysis emphasizes the importance of *PRR* as an indicator of hydrological function and resilience in CMAR. It highlights the need for ongoing monitoring of these ratios to understand their impact on water resource sustainability. The next section will explore *PWCR*, offering further insights into the intricacies of CMAR's water cycle.

#### 3.3.2 Precipitable water conversion rate (*PWCR*)

**Fig. 6:**
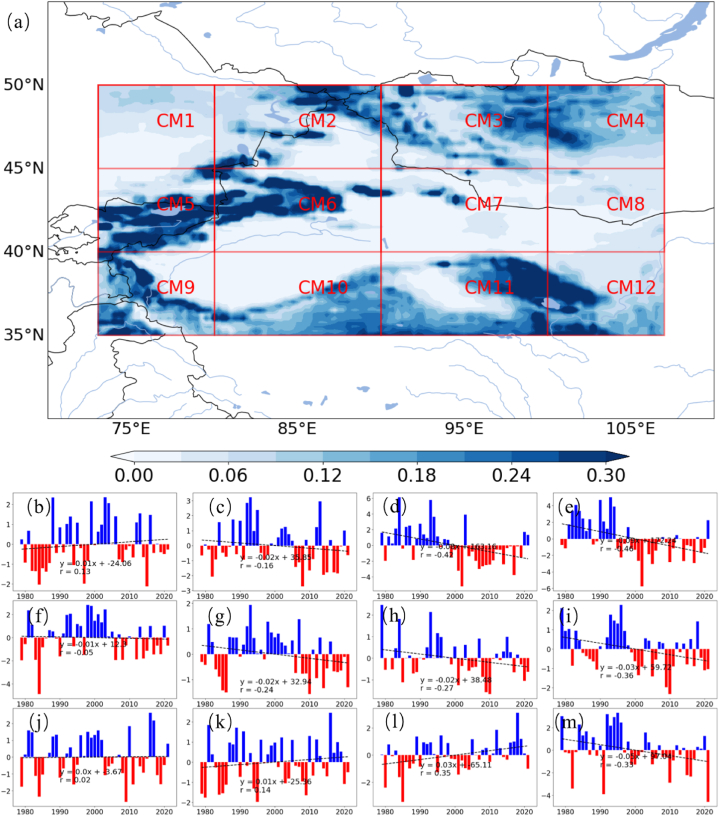
Spatial Distribution and Temporal Variations of Precipitable Water Conversion Rate (*PWCR*) (a) Spatial variation of *PWCR* across CMAR, with color intensity corresponding to conversion rates. (b–m) Temporal fluctuations of *PWCR* anomaly from 1980 to 2020 for each subregion. (For interpretation of the references to color in this figure legend, the reader is referred to the Web version of this article.)

Fig. 6 provides an analysis of *PWCR* across the subregions of CMAR. Fig. 6 (a) depicts the spatial distribution of *PWCR*, while Fig. 6(b–m) detail the subregions from CM1 to CM 12 annual changes from 1979 to 2021.

In Fig. 6 (a), a color gradient represents *PWCR* values, with darker shades indicating higher rates of atmospheric water conversion into precipitation. This distribution points to the influence of regional climatic factors conducive to precipitation events. Higher *PWCR* observed in subregions such as CM 2 and CM 6, as well as higher altitude areas (CM10, CM11) or regions with significant altitude variations (CM 9), suggests efficient atmospheric conversion processes, likely due to conditions favorable for convection.

Fig. 6(b–m) display bar charts illustrating the annual *PWCR* anomalies from CM1 to CM12. Blue bars represent years with increased conversion rates, possibly due to higher atmospheric moisture or favorable precipitation conditions, while red bars indicate years with reduced conversion rates, likely reflecting less conducive atmospheric conditions.

Certain subregions show clear trends over time. While the northwestern subregions (CM1) and the northern foothills of the plateau (CM10, CM11) exhibit increasing *PWCR* trends, other subregions predominantly show decreasing trends. This suggests that in the context of intensified advective transport (Fig. 4d), water vapor is not converted into precipitation but is instead transported to other regions.

This *PWCR* analysis, combined with the earlier examination of *PRR*, offers a comprehensive perspective on the intricacies of the water cycle in CMAR. Most subregions demonstrate a trend of increased evaporation with decreasing *PRR*, *PWCR*, and precipitation. The following section will integrate these findings using EOFs to further elucidate the hydrological interactions within the region.

## 4 Interplay of precipitation changes and water vapor dynamics: An empirical orthogonal function (EOF) exploration

This segment of the study explores the intricate relationship between *PRR* and *PWCR* on the hydrological cycle of CMAR, employing an Empirical Orthogonal Function (EOF) analysis for both variables.

### 4.1 Empirical orthogonal function analysis of PRR and PWCR

**Fig. 7:**
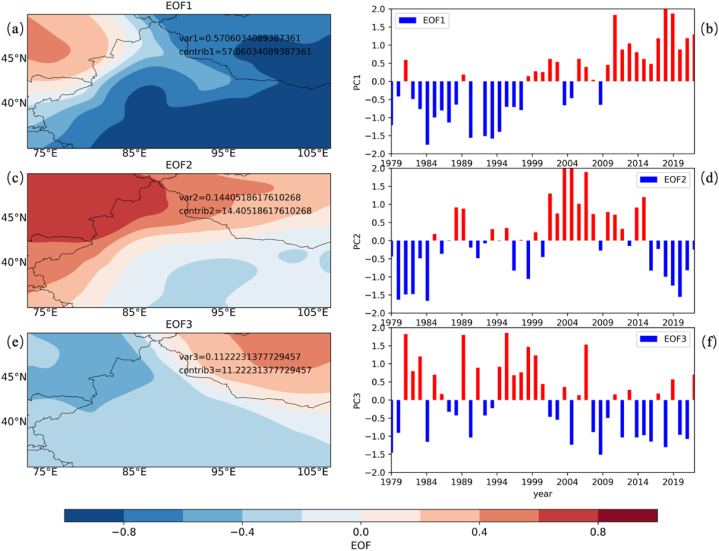
Empirical Orthogonal Function (EOF) Analysis of *PRR* in CMAR (a, c, e) Spatial patterns of the first three EOFs, representing dominant modes of *PRR* variability. (b, d, f) Temporal coefficients of the first three EOFs, showing interannual and decadal fluctuations in *PRR*.

Fig. 7 presents the results from an EOF analysis applied to *PRR* within CMAR. The analysis identifies the main modes of variability in *PRR*, with panels (a), (c), and (e) illustrating the spatial patterns of the first three EOFs and panels (b), (d), and (f) displaying their corresponding temporal variability.

EOF1, shown in panel (a) and its temporal pattern in panel (b), captures 57 % of the total variance, indicating its significant influence on *PRR* within CMAR. The spatial pattern reveals a dipole, suggesting contrasting recycling behaviors between the northern and southern parts of the region.

EOF2 and EOF3, represented in panels (c) and (e), account for an additional 25.6 % of the variance. The spatial pattern of EOF2 shows a gradient from north to south, while EOF3 presents an east-west division in *PRR* variability.

Combined, these three modes explain 82.6 % of the total variability, highlighting them as the principal factors influencing *PRR* dynamics in CMAR. The temporal coefficients, especially for EOF1, show variations that are likely tied to larger climatic patterns and regional atmospheric circulations.

The EOF analysis quantifies the significant spatial and temporal patterns in *PRR* variability across CMAR. The strong signal of EOF1 may be related to broad climate influences or specific regional water cycle feedback.

**Fig. 8:**
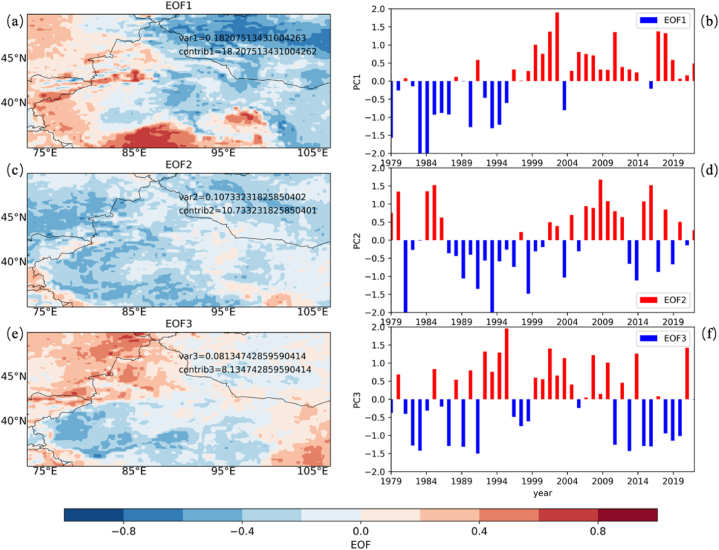
Empirical Orthogonal Function (EOF) Analysis of *PWCR* in CMAR, (a, c, e) Spatial distributions of the first three EOFs for *PWCR*, highlighting primary variability patterns. (b, d, f) Temporal progression of the EOFs from 1980 to 2020, depicting phases of variability in PWCR.

Fig. 8 conducts an Empirical Orthogonal Function (EOF) analysis of *PWCR* within CMAR. This analysis distinguishes the principal modes that contribute to *PWCR* variability. Panels (a), (c), and (e) showcase the spatial distribution of the first three EOFs, and panels (b), (d), and (f) detail their temporal progression from 1979 to 2021.

EOF1, contributing to 18 % of the total variance, exhibits a spatial pattern that transitions from positive to negative phases from southwest to northeast. This pattern indicates a southwest-northeast gradient in *PWCR* changes across CMAR. The temporal pattern of EOF1 is marked by alternating positive and negative phases, with a periodicity of approximately 5–10 years, indicating fluctuating trends in *PWCR* over time. The predominance of this mode underscores a notable pattern in the variability of *PWCR* within the region.

The subsequent modes, EOF2 and EOF3, collectively account for an additional 19 % of the explained variance, amounting to a cumulative total of 37 %. EOF2 displays a contrasting spatial relationship between the northern and central parts of CMAR, while EOF3 shows an east-west variation in *PWCR*.

This EOF analysis indicates that the variations in *PWCR* observed in CMAR are likely shaped by specific atmospheric circulation patterns or topographical influences, impacting the region's water vapor conversion dynamics. The analysis highlights the complexity of the hydrological processes in CMAR, suggesting a nuanced interaction between atmospheric conditions and the physical landscape.

Moving beyond the EOF analysis, the subsequent sections will correlate these modes with the physical processes that influence water vapor dynamics and precipitation patterns in CMAR, advancing our comprehension of the region's hydro-climatic interactions.

### 4.2 Spatial and temporal correlations

Comparing Fig. 7 (a, b) with Fig. 4 (c, d) reveals that the principal EOF of *PRR* shares commonalities with the spatial and temporal patterns of local water vapor changes (*∂q/∂t*). This comparison is not merely observational but speaks to an underlying physical link: variations in local water vapor are directly tied to shifts in the precipitation recycling process. Such local changes in water vapor within CMAR catalyze adjustments in *PRR*, subsequently affecting both the efficacy and quantity of precipitation. Fig. 7c and (e) illustrate the spatial patterns of the second and third EOFs, respectively, while Fig. 7d and (f) display their corresponding temporal variability. Both the second and third modes exhibit a periodicity of about 7–10 years, similar to the first mode, but each explains less than 15 % of the variance.

In a similar vein, when juxtaposing Fig. 8 (a, b) with Fig. 4 (e, f), it is evident that the primary EOF for *PWCR* aligns with patterns of advective water vapor changes (*∂qu/∂x* + *∂qv/∂y*). The correspondence between these figures suggests that the advection of water vapor is a key determinant of changes in the region's capacity for precipitation. The transit of water vapor across CMAR, subjected to diverse atmospheric and topographical influences, can amplify or reduce its precipitation potential, leading to the region's spatially varied precipitation distribution. Fig. 8c and (e) showcase the spatial distribution of the second and third EOFs, respectively, while Fig. 8d and (f) detail their temporal progression from 1979 to 2021. Although they exhibit similar patterns and changes to the first mode, the combined explained variance of the second and third modes is less than 20 %.

Such correlations underscore a dynamic interplay within CMAR: local water vapor alterations, driven by surface conditions and climatic factors, significantly influence the rate of precipitation recycling. Concurrently, the advection of water vapor, orchestrated by atmospheric circulation, is critical in determining the contribution of these air masses to CMAR's overall water budget.

The analysis thus far accentuates the critical role of these hydrological dynamics in the context of climatic variability. The responsiveness of CMAR's hydrological system to internal and external influences necessitates a focus on these interactions to comprehend current hydrological trends and forecast future shifts in the region's water availability.

## 5 Discussion

### 5.1 Interpretation of results

Our results provide a more nuanced understanding of the water cycle dynamics in CMAR, building upon previous findings that local and regional recycling processes contribute to about 10 % of the region's annual rainfall [5]. The spatial heterogeneity and temporal variability in *PRR* and *PWCR* revealed by our analysis highlight the complexity of these processes and the need for subregional-scale investigations to fully capture the intricacies of the water cycle in arid regions like CMAR.

The EOF analyses have elucidated that the first modes of both *PRR* and *PWCR* embody significant proportions of the total variance, with spatial and temporal correlations that underscore the delicate interplay between water vapor dynamics and precipitation. These modes reflect the influence of CMAR's topography and atmospheric conditions on hydrological variability. The results indicate that local changes in water vapor and the advection of moisture are closely intertwined with precipitation patterns, thereby shaping the region's hydrological characteristics.

Future research should focus on the following areas to further understand CMAR's hydrological dynamics: firstly, extended temporal analysis is crucial. Investigating hydrological trends and patterns over longer temporal scales could yield insights into decadal fluctuations and cyclical phenomena, particularly pertinent in the context of ongoing climate change. This approach would allow for a more comprehensive understanding of how long-term climatic variations influence CMAR's water cycle. Secondly, enhancing spatial resolution in hydrological studies is essential. Utilizing data with higher resolution could significantly refine the current understanding of hydrological processes at the subregional level. This would enable more granular insights into the interactions between local water vapor and precipitation, thus contributing to a more nuanced understanding of CMAR's hydrology. Thirdly, incorporating projections from climate models would be beneficial. This involves utilizing comprehensive climate models to simulate future climate scenarios, thereby aiding in predicting changes in CMAR's hydrological response to global warming and its associated climatic impacts. Fourthly, conducting detailed physical process studies is recommended. Such studies should focus on the underlying physical mechanisms driving the observed EOF patterns, including land-atmosphere interactions and the influence of orography. This would enhance the mechanistic understanding of the factors contributing to hydrological variability in the region. Fifthly, exploring atmospheric teleconnections is an important research direction. Examining the potential linkages between large-scale atmospheric circulation patterns and CMAR's hydrology could reveal how broader climatic forces shape the region's water cycle. Lastly, the application of advanced data assimilation techniques is suggested. These methods could effectively bridge the gap between observational data and simulation models, thus improving the accuracy and reliability of hydrological models for CMAR.

### 5.2 Implications for water resources management in CMAR

By addressing these areas, subsequent research can build upon the current findings, advancing the scientific comprehension of CMAR's water cycle dynamics. This progression is vital for predicting and potentially mitigating the impacts of environmental changes on this sensitive arid region's hydrological balance. While the findings of this study have implications for water resources management in CMAR and similar arid regions. Understanding the spatiotemporal variability of precipitation recycling and water vapor conversion can inform adaptive strategies to cope with water scarcity and climate change.

Given the observed trends of increasing evaporation and decreasing precipitation, PRR, and PWCR in most areas, water conservation and efficiency measures should be prioritized. This could involve advanced irrigation techniques, drought-resistant crops, and adjusted cropping patterns.

Insights into local moisture recycling and advective water vapor transport highlight the importance of land management practices. Maintaining vegetation cover in high PRR areas could enhance moisture retention and local recycling. Integrated watershed management approaches could optimize water allocation and minimize scarcity impacts.

Given the observed spatial heterogeneity in *PRR* and *PWCR* trends, it is crucial for studies of the CMAR region to focus on smaller sub-regions to avoid contradictory trends and conclusions drawn from topographic influences. This approach allows for a more nuanced understanding of the region's hydrological dynamics and ensures more accurate and effective water management strategies.

Effective water resources management in arid regions requires a holistic approach integrating scientific understanding with socio-economic considerations and stakeholder engagement. This study's findings provide a foundation for developing such integrated strategies, contributing to sustainable water management in the face of climate change and growing demands.

While this study focuses on CMAR, the methodologies and insights may be relevant for understanding hydrological dynamics in other arid regions. However, specific results, such as spatial patterns and temporal trends of *PRR* and *PWCR*, are likely to vary based on each region's unique topographical, climatic, and ecological characteristics. Factors like altitude, proximity to water bodies, wind patterns, and vegetation cover can significantly influence water cycle dynamics [55,56]. Therefore, while the general principles discussed may apply to other arid regions, the specific manifestations and magnitudes would require dedicated investigations accounting for the local environmental context. Future comparative studies across different arid regions could help elucidate common threads and unique features of water cycle dynamics in these water-scarce environments.

### 5.3 Limitations and future research directions

While this study focused on atmospheric moisture up to the 300 hPa level, it is worth noting that ERA5 does provide data at higher altitudes. Given that the study area is located in an arid region, most research confirms that the main moisture in arid regions is concentrated below 300 hPa [[57], [58], [59]]. Upper atmospheric moisture, although relatively low in concentration, can have important implications for the radiative balance and climate system in arid regions like CMAR. Water vapor in the upper troposphere contributes to both warming (through absorption and trapping of infrared radiation) and cooling (through emission of heat energy) effects [60]. Given the high elevation of certain areas within CMAR, such as the Tibetan Plateau, the role of upper atmospheric moisture may be particularly relevant. Future studies could explore the variability and trends of moisture at higher altitudes and their potential impacts on the hydroclimatic processes in CMAR.

While the subregions in this study are defined by consistent latitudinal intervals (5°), their longitudinal extents vary, resulting in subregions of different sizes (Table 1). This variation in subregion size is a consequence of the irregular shape of CMAR and the need to capture its spatial heterogeneity in terms of topography, climate, and hydrological processes.

Although the size of the subregions affects the absolute values of water cycle components, such as *PRR* and *PWCR*, this study primarily focuses on the trends and internal variations of these indices within CMAR. By conducting a subregional analysis, we aim to investigate the spatial and temporal variability of hydrological processes across the region, rather than comparing the absolute magnitudes of *PRR* and *PWCR* between subregions.

The subregional approach allows us to examine the relative changes and patterns of these indices within each subregion, providing insights into the localized hydrological dynamics and their responses to various environmental factors. This analysis helps to identify the subregions that exhibit distinct trends or variability in *PRR* and *PWCR*, contributing to a more comprehensive understanding of the water cycle within CMAR.

Furthermore, the EOF analysis employed in this study is designed to identify dominant modes of variability within the dataset, which are not dependent on the absolute sizes of the subregions. The EOFs capture the spatial patterns and temporal evolution of *PRR* and *PWCR* variability, providing insights into the underlying physical processes that drive the water cycle dynamics in CMAR.

Nevertheless, we acknowledge that future studies could explore the sensitivity of the results to different subregion delineations, such as using equal-area or hydrologically defined units, to assess the robustness of the findings presented here.

## 6 Conclusion

This study has systematically characterized the dynamics of the water cycle within CMAR through a meticulous analysis of ERA5 reanalysis data and simulations using the Dynamic Recycling Model (DRM). The results indicate that most areas in CMAR, from 1979 to 2021, have experienced a trend of increased evaporation, decreased *PRR* and *PWCR*, and reduced precipitation. To further analyze the underlying dynamics behind these changes, this study utilized methods such as Empirical Orthogonal Functions (EOFs) to examine the spatial and temporal characteristics of various water cycle variables in CMAR.

The EOF analysis identified that the first mode of *PRR* accounts for a considerable portion of the total variance, while the first mode of *PWCR* contributes a smaller, but still meaningful, percentage. These modes correspond closely with the observed spatial distributions and temporal variations in local water vapor changes (*∂q/∂t*) and advective water vapor changes (*∂qu/∂x* + *∂qv/∂y*). This finding underscores the strong connection between these vapor dynamics and the processes of precipitation.

Furthermore, the research demonstrates that the *PRR* and *PWCR* in CMAR are influenced by a combination of factors, including regional topography, atmospheric circulation patterns, and prevailing climatic conditions. The distinct spatial distributions and temporal trends revealed by the EOFs reflect the natural hydrological variability within the region.

In conclusion, this study has provided an in-depth analysis of the water cycle in CMAR, uncovering significant spatial heterogeneity and temporal variability. These findings highlight the sensitivity of CMAR's hydrological processes to both localized environmental changes and broader atmospheric dynamics, offering key insights into the region's natural hydrological mechanisms.

## Funding

This work was supported by the 10.13039/501100001809National Natural Science Foundation of China (No. 41801015), the 10.13039/501100004772Natural Science Foundation of Ningxia Province (2022AAC05065) and the 10.13039/501100002885China Meteorological Administration Innovation Development Special Project (CXFZ2024J043).

## Data availability statement

The data are available from the corresponding author on reasonable request.

## CRediT authorship contribution statement

**Ruolin Li:** Writing – original draft, Visualization, Methodology, Investigation, Formal analysis, Data curation, Conceptualization. **Qi Feng:** Writing – review & editing, Supervision, Resources, Project administration, Methodology, Funding acquisition, Conceptualization. **Yang Cui:** Writing – review & editing, Supervision, Funding acquisition.

## Declaration of competing interest

The authors declare that they have no known competing financial interests or personal relationships that could have appeared to influence the work reported in this paper.
