# Supplementary material for: Hydrological dynamics in the China-Mongolia arid region: An integrated analysis of precipitation recycling and water vapor conversion
Source: Heliyon. 2024 Jun 13;10(12):e32839. doi: 10.1016/j.heliyon.2024.e32839 (PMC11226913; doi:10.1016/j.heliyon.2024.e32839)
Supplement: Multimedia component 1 [file mmc1.docx]

Supplementary


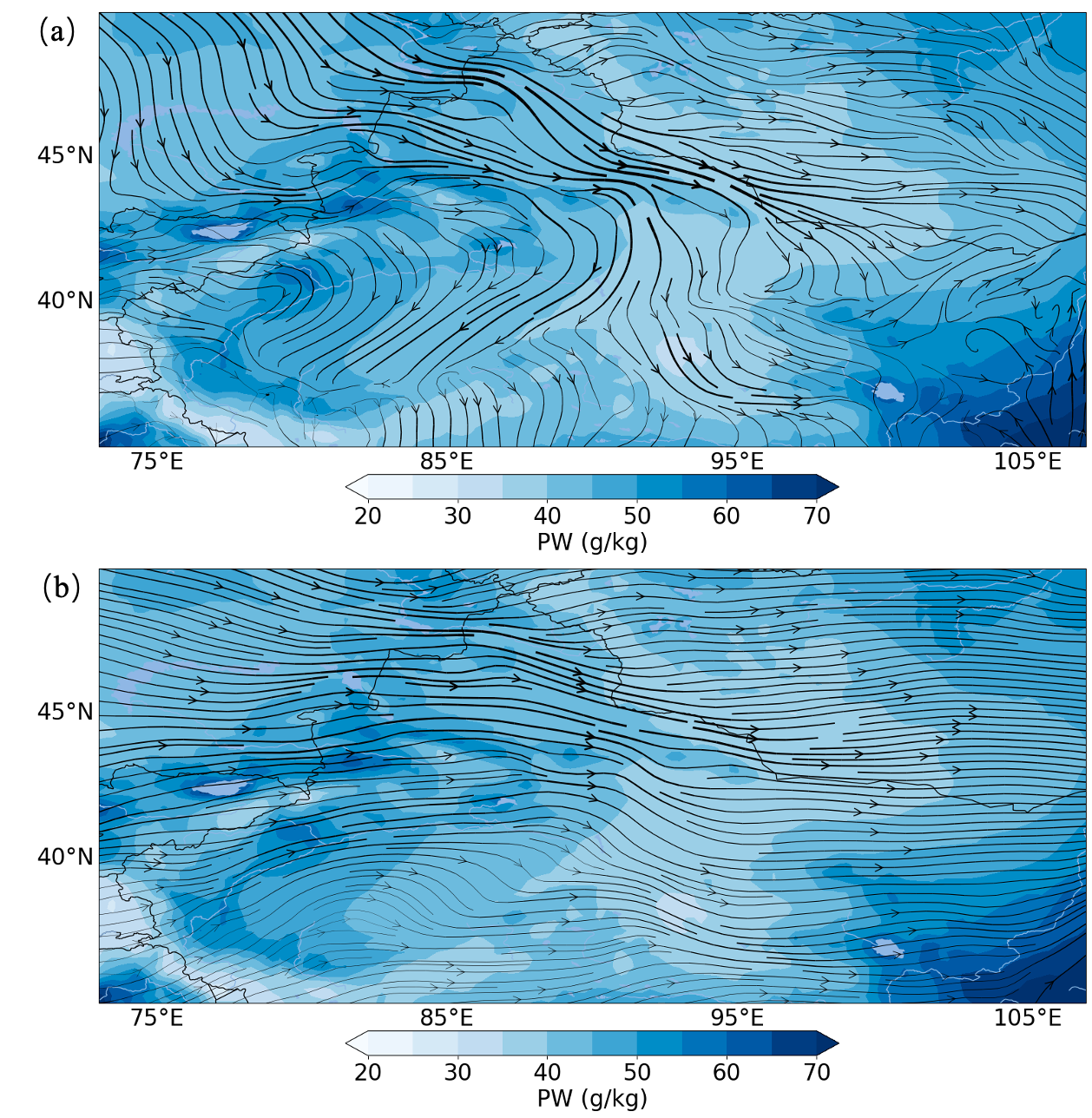


**Fig. S1** Comparison of Integrated Vapor Transport (IVT) values: (a) IVT calculated using Equation 1 and (b) IVT from direct ERA5 estimates. Both results show the westerly jet. The calculated IVT (a) better represents the influence of CMAR's topography on water vapor transport.
